# Modification of an automated precision farming robot for high temporal resolution measurement of leaf angle dynamics using stereo vision

**DOI:** 10.1016/j.mex.2025.103169

**Published:** 2025-01-13

**Authors:** Frederik Hennecke, Jonas Bömer, René H.J. Heim

**Affiliations:** a Institute of Computer Science, University of Göttingen, Goldschmidtstr. 7, 37077 Göttingen, Germany; b Institute of Sugar Beet Research, Holtenser Landstraße 77, 37079 Göttingen, Germany; c Institute for Geodesy and Geoinformation, University of Bonn, Nussallee 17, 53115 Bonn, Germany

**Keywords:** Leaf angle, Stereo vision, Functional traits, Plant phenotyping, Point cloud, 3D, Observing temporal leaf angle dynamics by retrofitting an open-source farming robot

## Abstract

In agriculture, the plant leaf angle influences light use efficiency and photosynthesis and, consequently, the overall crop performance. Leaf angle measurements are used in plant phenotyping, plant breeding, and remote sensing to study plant function and structure. Traditional manual leaf angle measurements have limited precision as they are labor- and time-intensive due to challenging environmental conditions and highly dynamic plant processes. To enable more detailed studies on leaf angles, we modified a well-established automated farming robot to obtain high-resolution 3D point clouds at customizable intervals of individual plants using stereo vision. We demonstrate the system's accuracy and reliability, with minimal deviation from reference values. The method can be utilized by other researchers to gather data on leaf angles and other structural plant traits at regular intervals to access the dynamics of leaves, plants, and canopies. The system's low cost and adaptability can enhance the efficiency of crop monitoring in plant breeding and phenotyping experiments. Detailed documentation and code are available on GitHub.

• An open-source farming robot is retrofitted to function as an automatic data collection platform
• Hard to access leaf angles can be retrieved with high accuracy
• Leaf angle dynamics can be observed with high temporal resolution

## Graphical abstract

**Figure:**
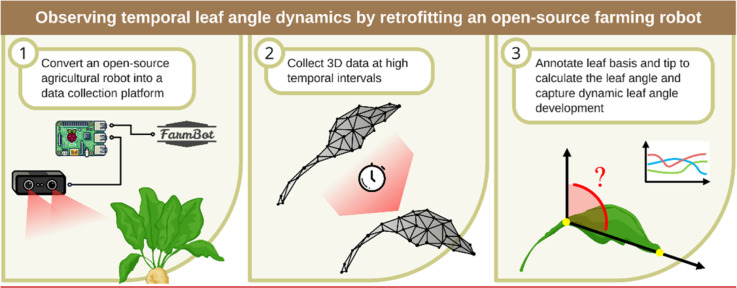

Specifications table

**Table:** 

Subject area:	Agricultural and Biological Sciences
More specific subject area:	Plant Phenotyping
Name of your method:	Observing temporal leaf angle dynamics by retrofitting an open-source farming robot
Name and reference of original method:	Not applicable
Resource availability:	• Farming Robot (FarmBot) • Stereo Camera (Intel RealSense D405) • Single Board Computer (Raspberry Pi 5) • Software (PointCloudHarvest)

## Background

The angle of a leaf represents a fundamental plant trait that influences a number of important physiological processes, including photosynthetic efficiency, light capture, and overall plant health [1]. This is a key driver for studies in ecophysiology, ecosystem ecology, agriculture, and remote sensing [2]. The dynamic distribution of leaf angles is a significant factor in the observation of vegetation from satellites, as it alters the reflectance properties of the canopy [3]. Therefore, accurate measurement of leaf angles is essential for understanding plant responses to environmental factors [4,5]. However, traditional methods of leaf angle measurement, such as the use of manual protractors or digital inclinometers, are labor-intensive, time-consuming, and require significant physical effort, often under difficult field conditions. The limitations of these methods preclude the possibility of obtaining a large sample size at a given timeframe and pose the risk of damaging the plants.

Alternative methods for leaf angle measurement have been explored to address these challenges. They utilize 3D models derived from laser scanning, digital photogrammetry, and various forms of 3D sensors to analyze the 3D structure of plants and leaves [[6], [7], [8]]. Müller-Linow et al. [6] have highlighted the dynamic nature of leaf angles of sugar beet in natural environments, where plants exhibit significant movement and angle changes over short periods. Their work demonstrates the need for frequent monitoring to capture these dynamics accurately. While the presented techniques offer improvement over manual methods and can generate high throughput using mobile robotic platforms, they still face limitations in terms of high costs and complex handling. Zhang et al. [9] summarizes current 3D data collection approaches for accessing the leaf angle of plants. As most of these require active human control, none seems effective at capturing dynamic changes in leaf angles . However, leaf angles are known to change over the course of a day and can be an indicator of multiple rapidly changing environmental factors like water stress [10].

To address these limitations, the introduced method utilizes an open-source farming robot as a low-cost platform for automated high-temporal 3D data collection and subsequent determination of the leaf angle of crops. The utilized farming robot is an open-source, automated platform known for its precision and minimal human intervention in various agricultural tasks. While it can autonomously seed, water, and monitor plants, its potential in detailed temporal plant phenotyping, particularly in measuring leaf angles, is not explored. Additionally, the required materials and code modifications of the platform needed for phenotyping applications are not available.

The primary objective of this study is to develop an automated method for accurately accessing the leaf angle of plants at a high temporal resolution for applications in commercial plant phenotyping and small to medium-scale research. Therefore, a new module for the farming robot was developed that uses a stereo-vision camera to automate the capture of 3D point clouds. The combined system of the farming robot and the stereo module enables to scan plants under outdoor growing conditions at short intervals for an extended period. This system offers high spatial accuracy through a gantry-based robotic configuration, while remaining cost-effective by employing consumer-grade technology. The proposed method is comprised of the following steps:

• Hardware: Build and integrate a stereo-vision camera module with the existing FarmBot hardware and software systems
• Software: Generate point clouds from recorded depth images using a downstream reconstruction pipeline
• Leaf angle extraction: Access the leaf angle using open-source software for point cloud processing

This project is documented in detail on GitHub, where the code and resources are available for public access.

## Method details

### Farming robot system

**Fig. 1:**
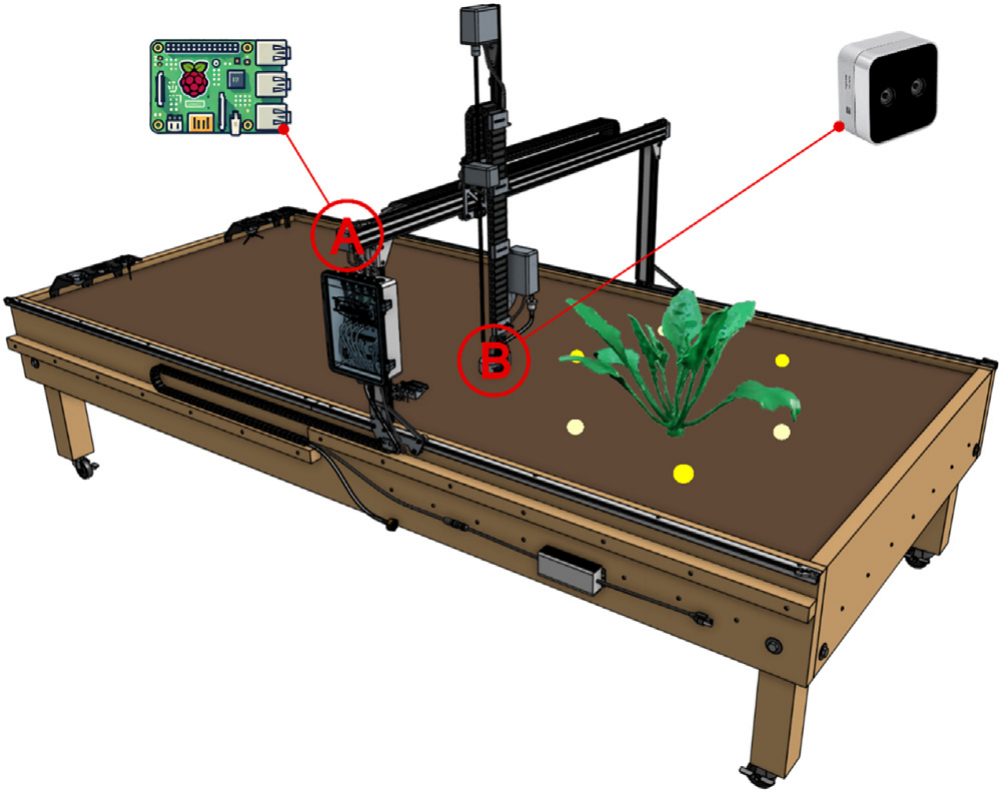
The FarmBot Genesis v1.6 was upgraded with a Raspberry Pi (A) to control the position (e.g., yellow dots) and interval of image capture events of an Intel RealSense D405 (B) depth camera. The camera was mounted at the bottom of the z-axis extrusion, while the associated Raspberry Pi was mounted above the electronic box on the gantry of the FarmBot. The camera generated 3D point clouds that served as a source for leaf inclination angle (LIA) retrieval. The accuracy of LIA measurement was validated using a 3D-printed reference model (green) with known LIA´s.

The method uses the FarmBot system (FarmBot Genisis v1.6, FarmBot Inc) (Fig. 1) as a robotic basis for automated data collection using an additional 3D sensor. The FarmBot is an open-source farming robot capable of executing a variety of agricultural operations with minimal human intervention in indoor and outdoor scenarios. The system is based on a hardware platform with a gantry-based robotic arm that can move in a 3D cartesian coordinate system. This enables the tool head to be positioned at varying points within a designated cultivation zone to interact with plants.

The FarmBot is mechanically controlled by a Raspberry Pi single-board computer (SBC), later called system SBC, that interfaces with various sensors and actuators. It is managed via a web interface that allows users to specify the position and variety of individual plants in the cultivation zone and to automate tasks such as planting, watering, and weed removal through exchangeable tool heads. Additionally, the system allows custom software in the form of LUA [11] scripts to be executed over the web interface.

### Stereo-vision camera

A stereo camera (RealSense D405, Intel Corporation) was utilized to collect high-precision 3D data. Stereo vision enables the reconstruction of a three-dimensional scene through the analysis of disparities between two or more images captured from slightly different viewpoints. The process mimics the functionality of human binocular vision [12]. The method is based on the identification of corresponding points in each image pair, with depth information being calculated based on their relative positions [13].

### Stereo module setup

A self-contained stereo module was developed to use the FarmBot system for high temporal 3D data collection. The module consists of the previously described stereo camera combined with an additional SBC (Raspberry Pi 5, Raspberry Pi Foundation) with a universal asynchronous receiver/transmitter (UART), later called stereo SBC. The function of the stereo SBC is to operate the stereo camera, which generates a considerable amount of data that cannot be processed by the system SBC on its own. This setup is also recommended in the FarmBot documentation [14].

**Fig. 2:**
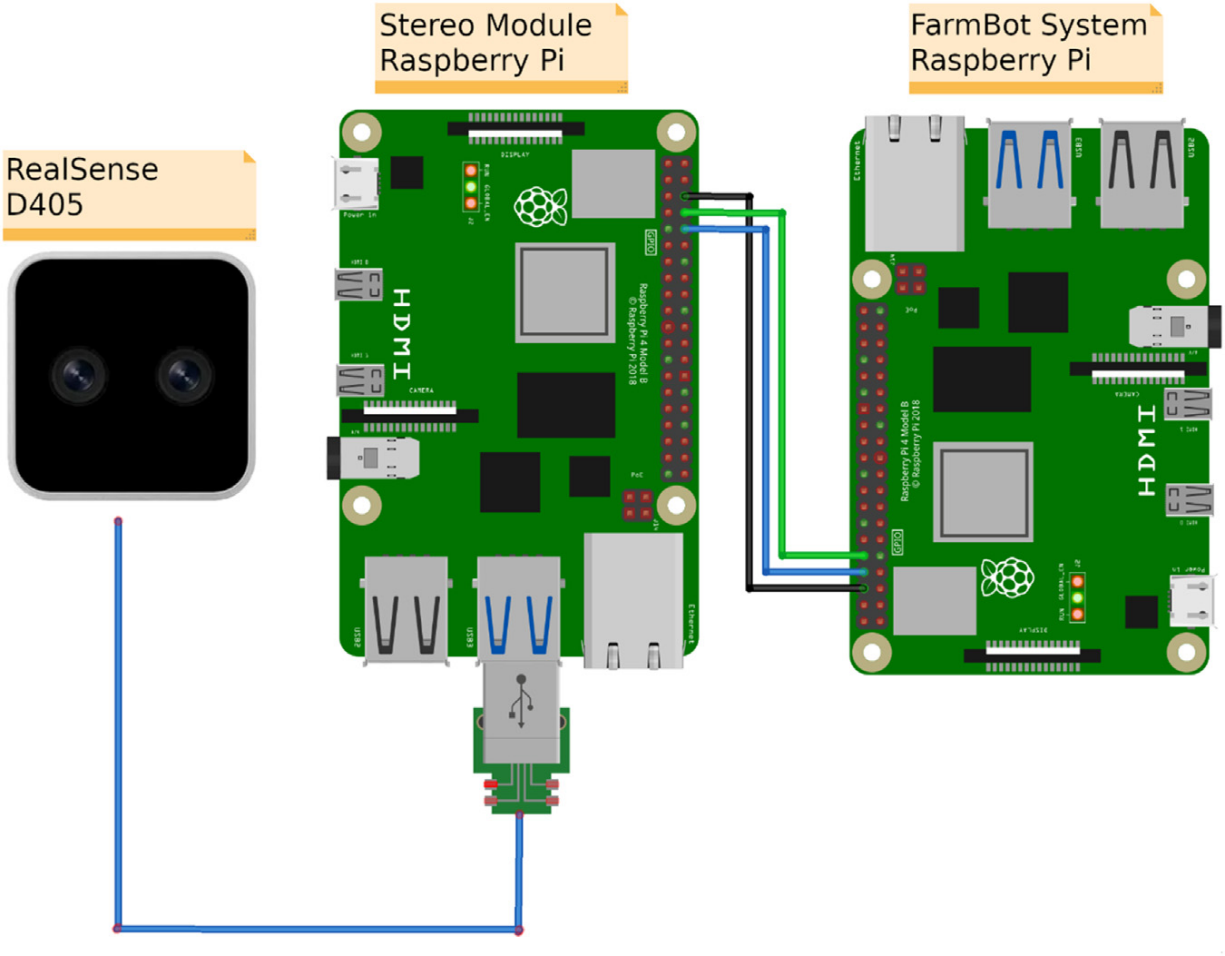
Hardware setup of the proposed stereo module. The FarmBot system SBC is connected to the stereo SBC via UART; The black cable is the ground connection, and the green and blue cables connect the receiver and transmitter pins. The stereo camera is connected via USB.

The camera and the stereo SCB were linked via a USB connection, while the stereo SBC and the system SBC were connected via UART to communicate and interact (Fig. 2). The stereo camera was mounted on the *z*-axis of the gantry arm, allowing it to move and capture images of single plants from different positions within the cultivation zone (Fig. 1). The stereo SBC was mounted next to the systems electronics box, which later allows for easy integration into the waterproof electronic system of the FarmBot. Both stereo module parts were secured using custom designed 3D-printed mounting brackets.

Besides the hardware integration, the necessary software for the communication of both SBCs and the control of the stereo camera was developed. The software is split into two different parts:

• A Lua script, which was executed using the web interface of the FarmBot to trigger data recordings. The script allows the FarmBot to communicate with a connected UART device, in this instance, the stereo SBC. Moreover, it provides metadata (plant ID and time stamp) of the recording to the stereo SBC. Using the "Events" tab on the FarmBot web interface, the script's execution intervals can be adjusted. The speed of the gantry and the radius and number of viewpoints can be customized and adjusted to different crops and growth stadiums.
• A Python script, which was executed on the stereo SBC to record data. The script waits for a UART signal and starts/stops the recording as soon as a signal is received. The recorded depth images are saved to a specified file path.

Detailed instructions for replicating the modified experimental setup presented in this study, as well as instructions for proper commissioning and use, can be found in the GitHub repository.

### Data collection

**Fig. 3:**
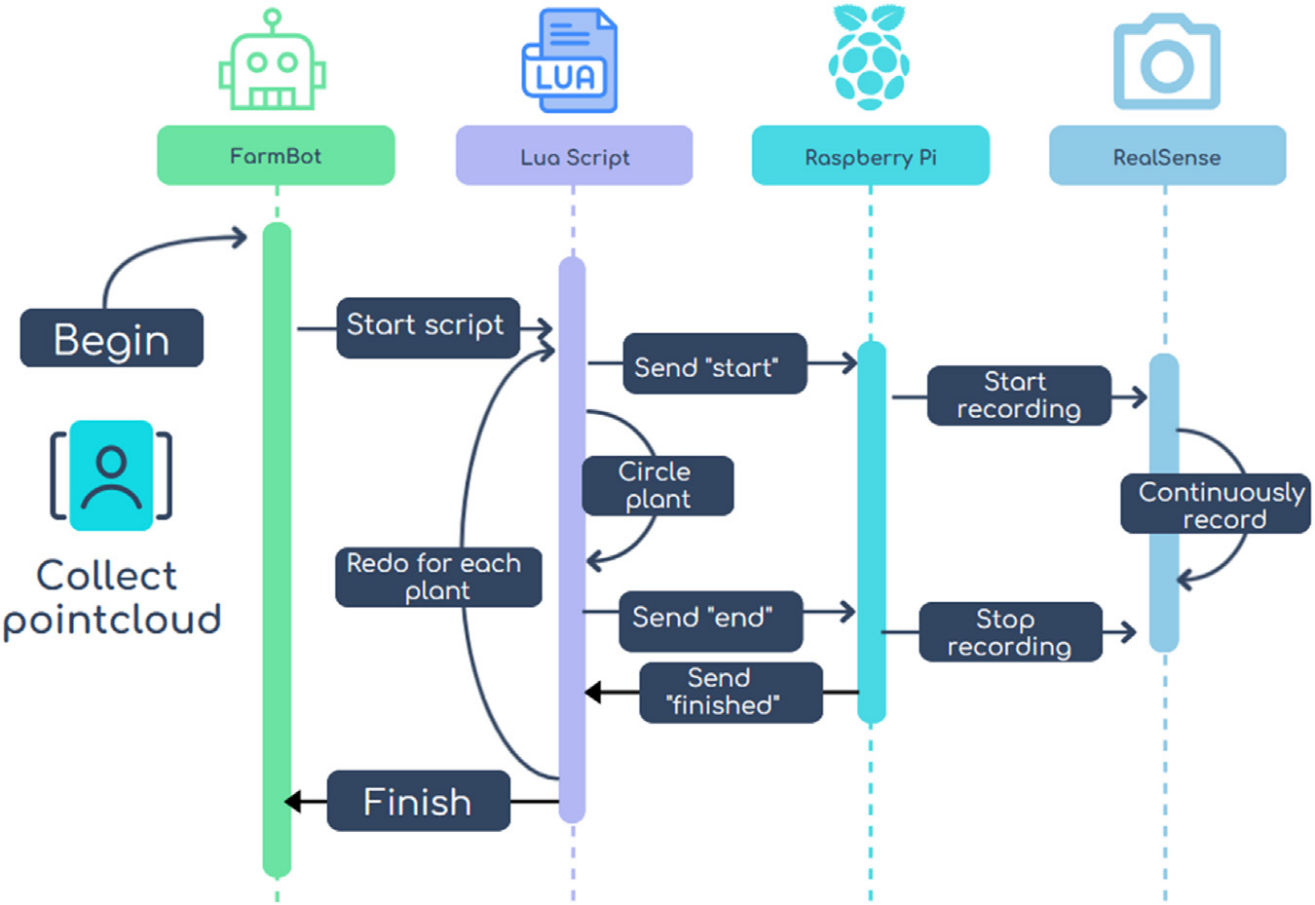
Software setup of the system SBC and the stereo SBC for data recording.

Once the FarmBot system is set up and plants have emerged or are positioned in the cultivation area, the data collection process can begin. Besides the usual preparations for starting up the FarmBot, the process involves the following steps:

• The location of the plant of interest in the cultivation area of the FarmBot needs to be set in the web interface of the FarmBot system and arranged into one plant group
• The Lua script needs to be set up with the help of the "Events" tab in the web interface to collect image data in defined intervals with a defined number of viewpoints. The following steps are undertaken during the execution of the script (Fig. 3):
  ○ Moving the gantry to the next plant in the specified plant group
  ○ Navigating between the calculated viewpoints for the plant while the stereo camera records data
  ○ Storing the images in a specified file path for further processing

### 3D reconstruction

The 3D reconstruction system creates a 3D point cloud from the previously recorded images. This script is a modified version of the Open3D reconstruction pipeline [15]. The pipeline is based on the approach by Choi et al. [16], with several modifications inspired by Park et al. [17] to enhance the reconstruction results. The reconstruction system in Open3D uses the previously taken depth images and camera intrinsics to generate a 3D model by aligning and integrating multiple frames into a coherent point cloud. It uses voxelization, pose graph optimization, and SLAC integration to enhance the model's accuracy, reduce noise, and ensure a consistent and reliable reconstruction. For further details about the workflow and adjustable parameters of the reconstruction system, we refer to [15].

Although the stereo module is capable of reconstructing 3D point clouds directly on the module itself, the reconstruction system was executed on a separate desktop PC due to the considerable computational demands of the process. This allows for the reduction of the system's data collection interval.

### Leaf angle extraction

Finally, the reconstructed point clouds can be loaded into CloudCompare [18], an open-source point cloud processing software. The leaf angle can be accessed for each individual leave of the recorded plant and for each point cloud in the timeline using the “Point picking list”-tool. The tool is used to annotate the points of the leaf blade basis and the leaf blade tip and extract their Cartesian coordinates. Subsequently, the leaf angle is calculated as the angle between the *z*-axis and a straight line connecting the leaf blade basis and the leaf blade tip:

1. Compute the magnitude of the vector from the blade basis to the blade tip
  $$\left|\vec{v}\right|=\sqrt{{\left(x_{\text{tip}}-x_{\text{base}}\right)}^{2}+{\left(y_{\text{tip}}-y_{\text{base}}\right)}^{2}+{\left(z_{\text{tip}}-z_{\text{base}}\right)}^{2}}$$
2. Compute the projection of $\vec{v}$ onto the z-axis
  $$v_{z}=z_{tip}-z_{base}$$
3. Compute the leaf angle θ
  $$\theta =\arccos\left(\frac{v_{z}}{\left|\vec{v}\right|}\right)$$

## Method validation

**Fig. 4:**
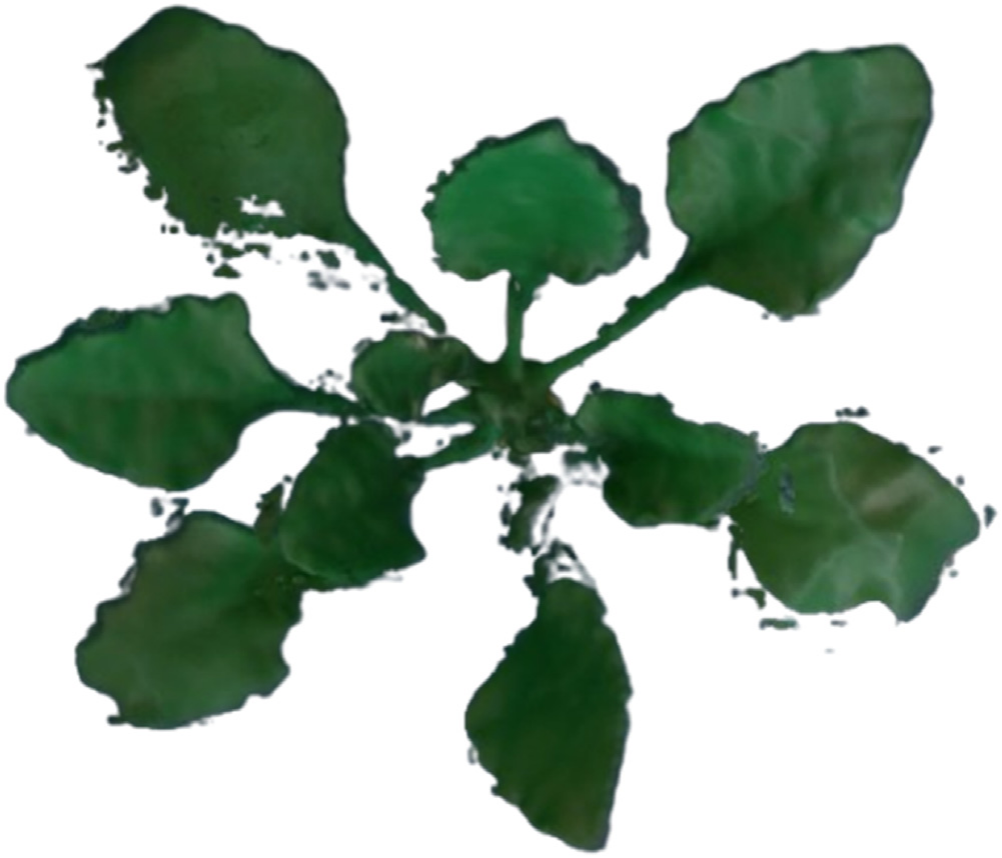
3D reconstructed mesh of the sugar beet reference model from Bömer et al. [19].

A 3D-printed reference plant of sugar beet was employed to assess the leaf angle extraction accuracy of the proposed method according to Bömer et al. [19]. Thereby, the accuracy of the proposed method was accessed by comparing automated digital measurements with manually collected benchmark data. The reference model was placed in the cultivation area (Fig. 1), and reference data was recorded three times in intervals of 15 minutes to demonstrate the high temporal data collection ability of the proposed method. Moreover, the data collection process was repeated three times with different numbers of viewpoints of the stereo camera arranged in a circle around the reference model. This allows for an analysis of reconstruction accuracy and ultimately leaf angle assessment accuracy of the proposed method depending on the number of viewpoints. An example of a reconstructed mesh from a captured point cloud is depicted in Fig. 4. The figure demonstrates the system's ability to accurately reconstruct significant portions of the reference model, with the exception of the two innermost leaves, which could not be reconstructed. Additionally, small artifacts surrounding the leaf edges are visible. Nevertheless, the two critical points for leaf angle assessment, the leaf blade basis and the leaf tip, are present for the majority of leaves, allowing the assessment of the leaf angle despite the presence of reconstruction artifacts.

The obtained leaf angle measurements demonstrate a high degree of accuracy of the proposed method. The differences between the manual reference measurement and the stereo module are low for a high number of leaves, indicating that the stereo-vision camera can provide reliable leaf angle data. The mean angle of three measurements for each leaf of the reference model can be seen in Table 1. Additionally, the table shows the results for three different reconstructions, each with a different number of viewpoints. In addition to the generally high accuracy of the measurements of all three measurement types with different number of viewpoints, it can be observed that the measurements with only one viewpoint exhibit the greatest discrepancy from the manual reference values for individual leaves. Furthermore, three leaves could not be measured at all due to high occlusion or defective reconstruction (leaf 2, 3, 6). This also applies to all measurement types carried out for leaves 11 and 12. The employment of three viewpoints is demonstrated to enhance the precision of leaf angle measurements and reduce occlusion, thereby rendering the three previously unmeasurable leaves measurable. By employing six viewpoints, the accuracy of leaf angle assessment improves again, but is comparable to the results for utilizing only three viewpoints. In summary, the measurement utilizing six viewpoints shows the lowest deviation from the manually collected reference values (Table 1).

**Table 1:** Leaf angle measurements in degrees for each leaf of the used reference model with an additional increasing number of viewpoints for 3D reconstruction. N = 3.

Viewpoints	Leaf											
	1	2	3	4	5	6	7	8	9	10	11	12
1	80°	-	-	45°	21°	-	46°	27°	17°	7°	-	-
3	96°	56°	72°	42°	23°	45°	42°	30°	16°	9°	-	-
6	100°	62°	75°	41°	22°	46°	42°	31°	16°	9°	-	-

Reference	104°	63°	85°	42°	20°	49°	41°	30°	18°	12°	10°	4°

**Fig. 5:**
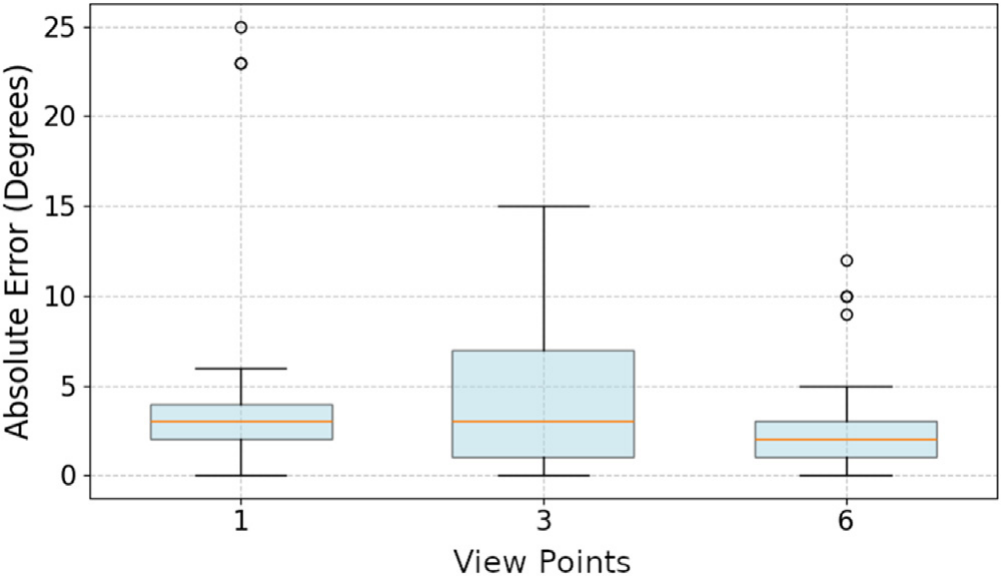
Measurement error in degrees of the leaf angles of the reference model depicted for three 3D reconstructions with different numbers of viewpoints.

The statistical analysis of the differences between the conducted measurements and the manual reference values showed that most leaf angle measurements fell within a small margin of error (Fig. 5). The measurement method utilizing six viewpoints exhibited the lowest mean margin of error (2.39°), while the other two methods with three (3.02°) or respectively one viewpoint (7.10°) were found to have similar levels of mean accuracy but exhibited a higher degree of measurement dispersion.

The validation proves the methods capability to measure leaf angles in sugar beet plants accurately by integrating a stereo-vision module into the FarmBot. The created point clouds enabled us to obtain accurate leaf surfaces, resulting in precise angle measurements. Minor differences between the measurements of the stereo-vision camera and the reference values confirm the accuracy of the developed system. However, it is noteworthy that the accuracy of the measured leaf angles increases with an increase in the number of viewpoints. This may be due to the reduction in occlusion of the model with an increased number of viewpoints, which results in the generation of more complete point clouds of the model and, consequently, more accurately measured leaf angles.

This is supported by the observation that, with a single viewpoint, three additional leaves could not be measured compared to the measurement methods with multiple viewpoints (Table 1). As illustrated in Fig. 5, the measurement method utilizing six viewpoints exhibits the lowest absolute measurement error across all measurable leaves of the reference model. However, with the use of more viewpoints for reconstruction, the time needed for recording a single plant rises. In consequence, there is a tradeoff between the targeted point cloud accuracy and the time needed for recording a 3D representaion of a single plant. Even though, utilizing six viewpoints per single plant is estimated to represent an effective balance between accuracy and the time required for data collection. It should be noted that this number may vary depending on the specific crop under analysis and the growth stage of the crop.

It is also important to note that, regardless of the number of viewpoints, the two innermost leaves of the reference model could not be measured due to their insufficient reconstruction. These findings align with those of Bömer et al. [19], who conducted a sensor comparison for 3D plant phenotyping and observed a similar phenomenon with photogrammetric reconstructions and LiDAR sensor data. It can thus be concluded that even modern 3D sensors, in combination with state-of-the-art 3D reconstruction techniques, encounter difficulties in reconstructing the fine details of small and closely packed leaves. Nevertheless, with a mean error of 2.39°, the developed stereo module offers high accuracy for all fully developed leaves of sugar beet and the possibility of temporal highly resolved data collection for detailed analysis of leaf angle dynamics over the course of days.

## Limitations

Despite the successful implementation of the module, a number of limitations and challenges apply. One significant issue for data collection is the impact of environmental factors, such as lighting conditions and wind, on the accuracy of the stereo-vision camera. Fluctuations in illumination can result in the introduction of noise into the point clouds, while wind can cause leaf movement, leading to the generation of defective three-dimensional reconstructions and the subsequent acquisition of inaccurate measurements. These issues could be addressed by installing LED lighting to stabilize the lighting conditions or by replacing the stereo camera with alternative sensors, such as time-of-flight cameras. Furthermore, wind-related inaccuracies could be mitigated by integrating weather data from a wind sensor into the FarmBot system.

In addition to its utilization with sugar beets, the proposed stereo module and measurement method can be employed with other crops initially expected to be cultivated by the FarmBot, including lettuce, carrots, and strawberries. Limitations associated with the cultivation of other agricultural crops, such as wheat or maize, are applicable to their height, as they are likely to interfere with the operations of the FarmBot's gantry and the range of the depth sensor. However, the monitoring of tall growing agricultural crops is also feasible during their early growth stages, prior to the development of their full height. When considering these limitations, the FarmBot system proposes an affordable phenotyping platform for all commonly used agricultural crops. Therefore, the application range of the proposed method extends from small-scale plant phenotyping platforms to larger phenotyping systems encompassing multiple FarmBot units. Another technical challenge is the processing time required for generating and analyzing point clouds. Although the recording system is relatively time-efficient, with around 45 seconds required to record data of a single plant using six viewpoints, further optimization of the code could improve processing speed. For full automation of the 3D reconstruction process directly on the stereo SBC, a more powerful SBC would be necessary to handle the increased computational demands.

Further research could concentrate on the automation of the system by implementing plant and leaf segmentation algorithms, as presented by Müller-Linow et al. [6], Yang et al. [2], and Itakura and Hosoi [8]. The implementation of these algorithms would facilitate the precise identification and isolation of individual plants and leaves, thereby enhancing workflow automation. Furthermore, the development of automatic parameter extraction on a single-leaf basis, utilizing techniques such as the template matching method presented by Marks et al. [20], would not only enhance the accuracy of multiple leaf angle measurements but also expand the system's phenotyping applications, including the monitoring of leaf area and the detection of diseases and their progression over time.

## Ethics statements

Not applicable.

## Supplementary material *and/or* additional information [OPTIONAL]

Not applicable.

## CRediT authorship contribution statement

**Frederik Hennecke:** Conceptualization, Methodology, Investigation, Visualization, Software, Writing – original draft, Writing – review & editing. **Jonas Bömer:** Conceptualization, Methodology, Supervision, Writing – review & editing. **René H.J. Heim:** Conceptualization, Methodology, Funding acquisition, Project administration, Supervision, Writing – review & editing.

## Declaration of competing interest

The authors declare that they have no known competing financial interests or personal relationships that could have appeared to influence the work reported in this paper.

## Data Availability

All used codes and recorded data are available at: https://github.com/FrederikHennecke/PointCloudHarvest.
